# Sympathetic activity in breast cancer and metastasis: partners in crime

**DOI:** 10.1038/s41413-021-00137-1

**Published:** 2021-02-05

**Authors:** Francisco Conceição, Daniela M. Sousa, Joana Paredes, Meriem Lamghari

**Affiliations:** 1 I3S—Instituto de Investigação e Inovação em Saúde, Universidade do Porto, 4200-135 Porto, Portugal; 2 INEB—Instituto Nacional de Engenharia Biomédica, Universidade do Porto, 4200-135 Porto, Portugal; 3 ICBAS—Instituto de Ciências Biomédicas Abel Salazar, Universidade do Porto, 4050-313 Porto, Portugal; 4 IPATIMUP—Instituto de Patologia e Imunologia Molecular da Universidade do Porto, 4200-135 Porto, Portugal; 5 FMUP—Faculdade de Medicina da Universidade do Porto, 4200-319 Porto, Portugal

**Keywords:** Bone cancer, Neurophysiology, Bone cancer

## Abstract

The vast majority of patients with advanced breast cancer present skeletal complications that severely compromise their quality of life. Breast cancer cells are characterized by a strong tropism to the bone niche. After engraftment and colonization of bone, breast cancer cells interact with native bone cells to hinder the normal bone remodeling process and establish an osteolytic “metastatic vicious cycle”. The sympathetic nervous system has emerged in recent years as an important modulator of breast cancer progression and metastasis, potentiating and accelerating the onset of the vicious cycle and leading to extensive bone degradation. Furthermore, sympathetic neurotransmitters and their cognate receptors have been shown to promote several hallmarks of breast cancer, such as proliferation, angiogenesis, immune escape, and invasion of the extracellular matrix. In this review, we assembled the current knowledge concerning the complex interactions that take place in the tumor microenvironment, with a special emphasis on sympathetic modulation of breast cancer cells and stromal cells. Notably, the differential action of epinephrine and norepinephrine, through either α- or β-adrenergic receptors, on breast cancer progression prompts careful consideration when designing new therapeutic options. In addition, the contribution of sympathetic innervation to the formation of bone metastatic foci is highlighted. In particular, we address the remarkable ability of adrenergic signaling to condition the native bone remodeling process and modulate the bone vasculature, driving breast cancer cell engraftment in the bone niche. Finally, clinical perspectives and developments on the use of β-adrenergic receptor inhibitors for breast cancer management and treatment are discussed.

## Introduction

Under physiological conditions, the sympathetic nervous system (SNS) is involved in the so-called “fight-or-flight” response to acute stress. Upon perceiving threats to internal homeostasis, the SNS acts on multiple molecular and cellular processes throughout the body that ensure a coordinated adaptive response to different stressors. Physical mobility is boosted through an increase in heart and respiratory rates, as well as through energy mobilization from adipose tissue and the liver^1,2^. On the other hand, anabolic processes such as digestion, gastrointestinal motility and reproduction are hampered^2–4^. Sympathetic signaling is mainly achieved through peripheral release of norepinephrine (NE) by sympathetic nerve terminals or systemic release of epinephrine (Epi) into the circulation by the adrenal glands. These catecholamines are the endogenous ligands of α/β adrenoreceptors (α-AR, β-AR), which exhibit widespread expression in a multitude of cell types and tissues^5–8^. This family of receptors is composed of a total of nine G protein-coupled receptors (GPCRs): G_q_-coupled α_1A_, α_1B_, and α_1D_ ARs; G_i_-coupled α_2A_, α_2B_, and α_2C_ ARs; and finally, G_s_-coupled β_1_, β_2_, and β_3_ ARs.

Breast cancer is still a major socioeconomic issue and was the leading cause of cancer-specific death in women in 2018 (https://gco.iarc.fr/today/home). It is a highly heterogeneous disease that is usually characterized by estrogen receptor (ER), progesterone receptor (PR), and epidermal growth factor receptor 2 (HER2) status of the primary tumor. Advances in diagnostic and adjuvant therapies have increased the life expectancy of patients with breast cancer, but this condition remains incurable in later stages of disease progression^9^. Surgery and radiation therapy are the gold standards for the treatment of early-stage breast cancer, as are hormone therapy and the HER2-targeting antibody trastuzumab for HER2-positive cancers. Systemic administration of hormone therapy, targeted therapy, chemotherapy or a combination of these is usually the preferred treatment approach for late-stage metastatic breast cancer. However, the 5-year survival rate of women diagnosed with distant metastasis is 27% (https://www.cancer.org/cancer/breast-cancer). These treatments are still ineffective and commonly associated with toxic side effects; therefore, there is still a need for improved therapeutic options. A better understanding of the pathological processes through which breast cancer thrives in the host is of paramount importance to discovering new therapeutic targets.

In the past decade, the physiological mechanisms that govern the response to stress have emerged as potential therapeutic targets in breast cancer due to findings from several epidemiologic and preclinical studies^10–12^. In particular, the action of NE and Epi on their cognate receptors has raised important considerations regarding their role in breast cancer progression, analogous to observations in other bone-tropic cancers such as prostate cancer^13–17^. However, the adrenergic regulation of the multiple cellular processes that drive breast cancer remains a matter of intense debate.

In this review, we discuss the current knowledge found in the literature concerning preclinical and clinical data on SNS modulation of breast cancer. Most patients with metastatic breast cancer present severe skeletal complications such as hypercalcemia, pain, and an increased incidence of fractures^18^. Therefore, insight into the sympathetic regulation of bone metastatic disease is also discussed in the following sections.

## Breast cancer and the SNS: a complex picture

Adrenoreceptors (ARs) have been reported to be expressed in a wide range of breast cancer cell lines (Table 1) as well as in tumor samples from patients with breast cancer^19–21^. AR overexpression, particularly β_2_-AR overexpression, was found to be correlated with poor prognosis of ER^−^ breast cancer patients in a recent study by Kurozumi et al.^21^, where immune biomarkers, such as the grades of tumor-infiltrating lymphocytes and programmed death ligand 1 expression, were shown to be significantly reduced in these patients. Another report by Liu et al.^19^ demonstrated that the β_2_-AR level was correlated with lower disease-free survival and higher lymph node metastasis rates in a small cohort of HER2^+^ breast cancer patients. Both of these studies point to a putative role of β_2_-AR in breast cancer pathology, but scrutinizing the mechanisms by which it promotes disease progression is still a complex exercise. In this section, we assemble the available data regarding the effect of multiple ARs on breast cancer, from primary tumor proliferation and survival to extracellular matrix (ECM) invasion and entry into the systemic circulation.

**Table 1:** AR expression in human breast cancer cell lines

Cell line	Molecular subtype	AR(s) expressed	Reference
T47D	Luminal A (ER^+^, PR^+^, HER2^−^)	α_2A_-AR, α_2B_-AR, α_2C_-AR	^130^
MCF7	Luminal A (ER^+^, PR^+^, HER2^−^)	α_1_-AR, α_2B_-AR, α_2C_-AR, β_1_-AR, β_2_-AR	^19,50,131,132^
ZR-75	Luminal A (ER^+^, PR^+^, HER2^−^)	β_1_-AR, β_2_-AR	^131^
BT474	Luminal B (ER^+^, PR^+^, HER2^+^)	β_2_-AR	^19^
SKBR3	HER2 (ER^−^, PR^−^, HER2^+^)	β_2_-AR	^19^
MDA-MB-453	HER2 (ER^−^, PR^−^, HER2^+^)	β_2_-AR	^131^
MDA-MB-231	Basal (ER^−^, PR^−^, HER2^−^)	β_2_-AR	^11,47,132,133^
MDA-MB-468	Basal (ER^−^, PR^−^, HER2^−^)	β_1_-AR, β_2_-AR	^131,133^
HS578T	Basal (ER^−^, PR^−^, HER2^−^)	α_2A_-AR	^132^

### Proliferation and survival

**Fig. 1:**
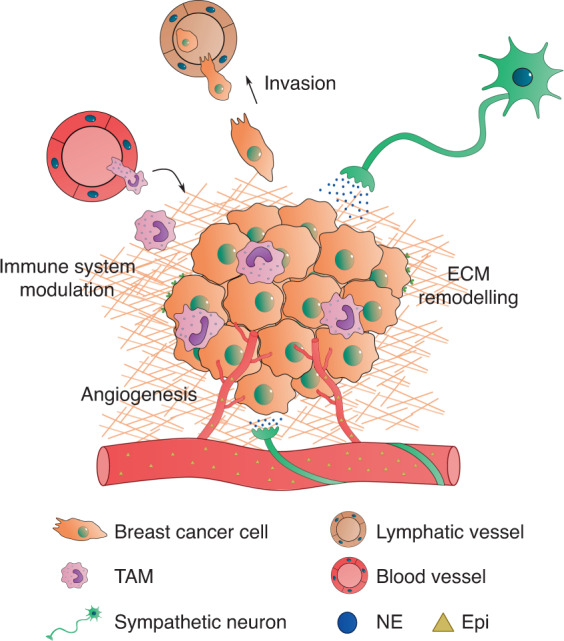
Sympathetic control of breast cancer progression. NE released from sympathetic neurons closely associated with blood vessels, as well as Epi that diffuses from the circulation, modulate several important hallmarks of breast cancer such as survival, angiogenesis, immune surveillance escape, ECM remodeling and invasion. NE, norepinephrine; Epi, epinephrine; TAM, tumor-associated macrophage; ECM, extracellular matrix

**Fig. 2:**
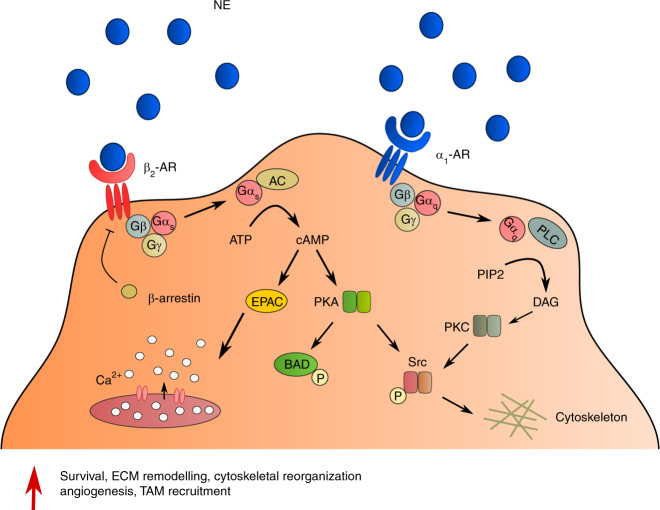
Adrenergic receptor downstream signaling. β_2_-AR activation triggers several downstream signaling pathways, mediated by an increase in intracellular cAMP, leading to Ca^2+^ release, apoptosis inhibition through phosphorylation of BAD, and cytoskeletal rearrangement. β_2_-ARs are quickly desensitized by β-arrestins after ligand binding and signal transduction. Alternatively, α_1_-AR stimulation has also been described to promote invasive phenotypes through PKC-mediated signaling pathways. NE, norepinephrine; TAM, tumor-associated macrophage; ECM, extracellular matrix

Cancer cell proliferation and apoptosis inhibition are crucial hallmarks of cancer^22^. Adrenergic signaling has been implicated in several apoptosis pathways, and it has been previously suggested that endogenous catecholamines directly exert prosurvival effects on breast cancer cells^23–25^ (Figs. 1, 2). Epi was described as an antiapoptotic stimulus in human breast cancer cells in vitro, inactivating the proapoptotic protein BAD through phosphorylation in a PKA-dependent manner^24^. Furthermore, another in vitro experiment by Reeder et al. showed that NE and Epi decrease the efficacy of commonly used drugs targeting proliferating cells, such as paclitaxel, since these catecholamines arrest MDA-MB-231 breast cancer cells in G1 phase, decelerating the cell cycle^25^. These results are consistent with evidence from other in vitro studies showing that β_2_-AR agonists inhibit triple-negative breast cancer cell proliferation and DNA synthesis^23,26,27^. Strikingly, low concentrations of Epi increased MCF7 and MDA-MB-231 cell proliferation, while the β_2_-AR agonist isoproterenol decreased the proliferation of both cell lines^27^. These findings could be explained by the observation that Epi was shown to differentially bind to distinct ARs depending on its concentration, with greater affinity for α_2_-AR at nanomolar concentrations and shifting to β_2_-AR binding at micromolar concentrations^23^. Moreover, the increase in proliferation evoked by low concentrations of Epi was abrogated by the addition of the α_2_-AR antagonist rauwolscine^23^. Exciting questions remain, such as the following: what is the impact of fluctuations in Epi or NE levels in the tumor microenvironment on breast cancer progression, and how can this knowledge be translated to a clinical setting? There is already recent in vivo evidence that sheds some light on the impact of circulating Epi on tumor growth; Walker and colleagues have shown that adrenal denervation and inhibition of Epi release do not impact disease progression^28^.

Some observations from in vivo studies point to a negligible effect of β-ARs on primary tumor growth, since compared to vehicle control treatment, isoproterenol stimulation of orthotopic breast cancer tumors did not change primary tumor proliferation^11,23,29,30^. It is unclear whether these results arose from the direct action of β_2_-AR on tumor cell proliferation, inhibition of tumor growth by other cell types in the stroma or even a combination of direct and indirect effects. Another study using human xenografts in immunocompromised mice reported increased ER^+^/PR^+^ breast cancer tumor growth after inoculation with the α_2_-AR agonist clonidine^31^. The increase in tumor growth was accompanied by a similar increase in the proliferation of tumor-associated fibroblasts, and thus, an indirect effect of α_2_-AR agonism through the tumor microenvironment cannot be ruled out^31^. It is also intriguing that Thaker et al. reported an increase in MDA-MB-231 tumor growth after chronic stress induction in an orthotopic breast cancer model^32^, contrasting with the studies previously discussed. Notably, pharmacological β-AR activation seems to inhibit primary tumor growth^11,23^, while endogenous chronic stress either causes negligible effects or increases tumor growth^29,30,32–34^. This observation raises important questions, such as whether compensatory mechanisms are exerted by other ARs in endogenous stress models, since Epi and NE can stimulate both α-ARs and β-ARs. In fact, α_2_-AR antagonists were shown to counteract the increase in tumor growth evoked by restraint stress^33^. Lamkin and colleagues also showed that in the absence of chronic stress, α_2_-AR blockade recapitulated the tumor growth observed when the SNS was endogenously activated^33^, adding another layer of complexity to the impact of SNS signaling on breast cancer. This effect probably arises because presynaptic α_2_-ARs in peripheral SNS neurons establish a negative feedback loop to control NE release from neuronal terminals^35^. Thus, blockade of α_2_-ARs in the absence of chronic stress increases the release of NE in the tumor microenvironment, mirroring endogenous activation of the SNS.

### Angiogenesis

As breast tumors proliferate and grow, the need for nutrients and oxygen rises concordantly. These needs are met by the sprouting of new blood vessels that give rise to a network of often aberrant vasculature in the tumor microenvironment^36^. The SNS has emerged as an important player in neoangiogenesis, since it has already been shown that sympathetic outflow can induce the secretion of proangiogenic factors, namely, vascular endothelial growth factor (VEGF), by breast cancer cells^29,37–39^. In addition, direct cell–cell contact between breast cancer cells and endothelial cells leads to increased formation of capillary structures in vitro, a result markedly potentiated by the addition of NE^38^. This effect was suggested to be mediated by the β_2_-AR/PKA/mTOR pathway and by upregulation of the Notch ligand Jagged-1, directly augmenting Notch signaling in endothelial cells^38^. Interestingly, there seems to be a cell-specific response to β_2_-AR agonists in terms of VEGF expression that is not entirely due to differential β_2_-AR expression^37^. β_2_-AR agonists were found to increase VEGF production in a brain-tropic variant of the MDA-MB-231 cell line in vitro but not in the parental cell line or in cells with low β_2_-AR expression, such as MCF7 cells^37^. Distinct targets of downstream effectors of the β_2_-AR/PKA pathway in the different cell lines might explain the disparity in terms of angiogenic responses.

Other players have recently been suggested to be involved in the sympathetic regulation of tumor angiogenesis. Activation of peroxisome proliferator-activated receptor γ (PPARγ) was shown to markedly decrease VEGF expression in 4T1 murine breast cancer cells in vitro, and NE was shown to inhibit PPARγ expression in these cells^39^. This inhibition was abrogated by the addition of ICI118551, pointing towards a β_2_-AR-mediated effect^39^.

In addition to the in vitro data previously discussed, accumulating evidence from several in vivo studies indicates that chronic stress modulates neoangiogenesis and the lymphatic vasculature in breast cancer. Chronic restraint stress, as a model of endogenous SNS activation, was found to increase VEGFC secretion from MDA-MB-231 orthotopic tumors in immunocompromised mice, as well as from 66cl4 tumors in immunocompetent mice, leading to increased tumor lymphatic vessel density^29^. This effect was recapitulated or abrogated by isoproterenol or propranolol treatment, respectively, suggesting the existence of a β-AR-specific signaling pathway^29^. Stress-induced production of VEGF in 66cl4 primary breast tumors in mice and a consequent increase in vascularization were also described^30^. The increased tumor vasculature was also suggested to be an additional route of cancer cell escape^29,30^ (Fig. 1), facilitating metastasis, as discussed in the following sections.

### Immune system modulation

The crosstalk between the SNS and the immune system in the regulation of inflammation is already recognized. Dendritic cells and monocytes express both the α-AR and β-AR subtypes, and adrenergic activation in these cells leads to downregulation of tumor necrosis factor α (TNF-α), IL-1, and IL-6, resulting in the promotion of an immunosuppressive phenotype^40^. The effect of the SNS on the different immune cell populations in the context of inflammation and hematopoiesis has already been previously reviewed^41^.

Among the many cellular components of the tumor microenvironment that are affected by SNS catecholamines, tumor-associated macrophages (TAMs) are crucial for cancer progression. SNS signaling prompts breast cancer cells to secrete proinflammatory cytokines, such as IL-6^37^ and M-CSF^30^, which can enhance the recruitment and infiltration of macrophages into the primary tumor (Fig. 1). On the other hand, β_2_-AR activation in macrophages increases the expression of cancer progression-promoting factors, such as transforming growth factor β (TGF-β), matrix metalloproteinase (MMP) 9, VEGF and cyclooxygenase-2 (COX2), in vivo^30^. Macrophage expression of COX2 and consequent secretion of prostaglandin E2 (PGE2) further drives the production of VEGFC by cancer cells to induce lymphangiogenesis^29^. In addition, in an orthotopic breast cancer model, peripheral sympathetic nerve ablation using 6-hydroxydopamine led to inhibition of TAM recruitment and to a decrease in tumor IL-6 levels^42^.

Upon chronic stress induction in syngeneic breast cancer mouse models, TAMs are mostly primed towards an immunosuppressive M2 phenotype: genes such as Arginase-1 and IL-10 are overexpressed, while M1 phenotype-characteristic genes are conversely downregulated^30,43^. In addition, Bucsek et al. reported a significant decrease in tumor-infiltrating effector cytotoxic CD8^+^ T cells upon β-AR activation and concomitant 4T1 breast cancer tumor growth^44^. Immunosuppressive CD4^+^ Treg cells and splenic myeloid-derived suppressor cells were also elevated in stressed mice^44^.

Furthermore, and in agreement with the reports discussed above, Kamiya et al. elegantly illustrated the influence of tumor sympathetic innervation on immune checkpoint expression and cancer progression^34^. With a viral vector-based tool, the authors were able to specifically denervate the tumor stroma without affecting surrounding tissues^34^. The subsequent decrease in tumor NE content abrogated tumor growth and metastatic spread. Moreover, sympathetic denervation downregulated immune checkpoint molecules, such as programmed death 1 (PD-1), in β_2_-AR-expressing CD4^+^ and CD8^+^ tumor-infiltrating lymphocytes. The authors observed the same outcomes in chemically induced and spontaneous breast cancer models and reported correlations between the density of sympathetic fibers, PD-1 expression and tumor recurrence in a small cohort of human breast cancer patients^34^.

These observations reinforce the hypothesis that SNS-driven immunosuppression and subsequent evasion of immune surveillance play an important role in breast cancer progression.

### Extracellular matrix invasion

As the disease progresses, a cascade of cellular events triggers the ability of breast cancer cells to remodel and invade adjacent tissues, eventually escaping into the circulation through intravasation into blood or lymphatic vessels^45^. Crosstalk between the tumor microenvironment and breast cancer cells is crucial for the acquisition of invasive features, and the SNS has been directly linked to the process of epithelial-to-mesenchymal transition (EMT)^46^.

Adrenergic signaling, namely, through β_2_-AR, has been shown to directly modulate several cellular processes in breast cancer cell lines. Isoproterenol stimulation led to increased invasive capacity of highly metastatic MDA-MB-231 cells in vitro, and this effect was β_2_-AR specific^46^. Interestingly, overexpression of β_2_-AR in MCF7 cells resulted in increases in the number of invadopodia and the invasive capacity after incubation with isoproterenol^46^.

The molecular mechanisms that govern this adrenergic response have begun to be elucidated in recent years. Stimulation of β_2_-AR in vitro causes the accumulation of intracellular cAMP through the Gα_s_/adenylyl cyclase pathway and consequent dephosphorylation of ERK1/2^47^. This increase in cAMP activates PKA and exchange protein directly activated by cAMP, leading to increased mobilization of Ca^2+^ in a feedforward loop that ultimately drives cell invasion mechanisms^47^ (Fig. 2). In other in vitro studies, β_2_-AR activation led to increased motility and invasiveness of MDA-MB-231 cells, partially through changes in actin remodeling and contractility and an increase in plasma membrane protrusions^48,49^. Interestingly, the β-AR agonist isoproterenol reduced the number of focal adhesions while increasing the number of invadopodia, favoring motility in three-dimensional spaces but not on two-dimensional surfaces^48^.

Although most of the available data in the literature are from experiments with β_2_-AR and MDA-MB-231 cells, other cell lines and ARs should not be overlooked. Dezong et al. reported that invasion and migration mediated by the proto-oncogenic tyrosine protein kinase Src were modulated by different ARs in the MDA-MB-231 and MCF7 cell lines in vitro, namely, β_2_-ARs and α_1_-ARs, respectively^50^. Src was found to be targeted for phosphorylation via different signaling pathways, i.e., PKA in MDA-MB-231 cells and PKC in MCF7 cells^50^. These data might explain the seemingly contradictory results observed in previous studies, where the migration capacity of MCF7 cells was described to be decreased upon stimulation with the β-AR agonist isoproterenol^27^. The same study reported a decrease in MDA-MB-231 cell migration after isoproterenol stimulation^27^, possibly because a parental MDA-MB-231 cell line was used instead of a highly metastatic variant of the MDA-MB-231 cell line^47–49^.

In addition to the direct effects of NE on breast cancer cells, stimulation of tumor stromal α_2_-AR was reported to promote breast cancer progression and invasion. Pharmacological activation of α_2_-AR but not α_1_-AR or β-AR increased the rate of metastasis in a syngeneic orthotopic breast cancer model^51^. These changes were correlated with altered collagen structure and were cancer cell independent, since the cell line used did not respond to NE in vitro^51^. However, no insight was provided on the stromal players targeted by α_2_-AR agonizts that are involved in collagen remodeling.

As can be appreciated by the collective results of previous studies, the interplay between breast cancer and the SNS is extremely complex. Clearly, knowledge concerning the combination of α-AR and β-AR signaling on cancer progression, as well as on the distinct cellular players in the tumor microenvironment, is still scarce. Therefore, careful consideration should be exercised when designing experiments and therapeutic interventions.

## Breast cancer metastasis and the bone niche

After escaping into the vasculature, breast cancer cells disseminate and travel towards distant organs in a complex multistep process that has not yet been fully elucidated. Breast cancer exhibits specific tropism for organs such as the lung, brain, liver and bone, and there are indications that this tropism is associated with breast cancer receptor status^52^. Luminal A/B tumors are the most prevalent subtype in patients with breast cancer, and they mostly metastasize to bone^53,54^. Luminal A/B bone metastases are typically indolent in the first years of follow-up, and patients presenting only bone metastases have higher overall survival rates than patients presenting metastasis to other distant sites^53,54^. However, ~70% of all late-stage breast cancer patients exhibit bone metastatic foci leading to severe complications such as hypercalcemia, pain and bone fractures^52,55^. Metastatic foci are found mostly in long bones, ribs, the pelvis, and vertebrae, which contain abundant marrow and provide an immune context favorable for cancer cell survival; the bone marrow microenvironment is crucial for the maintenance of the hematopoietic stem cell niche^56^. In addition, bone stromal cells secrete a combination of cytokines and growth factors that favor breast cancer cell homing, survival, and proliferation^57^. Breast cancer cells establish close interactions with bone cells, namely, osteoclasts and osteoblast-lineage cells, and the SNS can potentiate this crosstalk.

### The metastatic vicious cycle

The skeletal system plays a critical role in all stages of human development. The skeleton is responsible for locomotion; it is the preferential site for hematopoiesis, regulates mineral homeostasis and protects vital organs, such as the brain, heart and lungs. It is therefore crucial to maintain skeletal structural integrity and function throughout life. This maintenance is achieved mainly through a highly dynamic bone remodeling process, where the bone matrix is degraded and subsequently replaced by new mineralized bone in a coordinated fashion. Osteoclasts are specialized multinucleated cells of the hematopoietic lineage that are able to demineralize and resorb the bone matrix using a combination of secreted enzymes, such as cathepsin K (CatK)^58^ and tartrate resistant acid phosphatase^59^. During resorption, factors secreted from osteoclasts and byproducts of bone matrix degradation recruit precursors of bone-forming cells, coupling bone resorption and bone formation. These precursors of a mesenchymal lineage differentiate into mature osteoblasts, which are then responsible for the deposition of high amounts of ECM proteins and for their mineralization^60^. Osteoblasts can then entomb themselves in the matrix that they produce and transform into osteocytes. These cells account for more than 90% of the cells present in cortical bone and have long extensions, creating an interconnecting network between osteocytes themselves and cells in the bone marrow^61^. Osteocytes are thought to have an endocrine^62^ and mechanosensitive role^63,64^ in bone, participating in complex adaptations to internal and external stimuli.

Breast cancer often leads to highly osteolytic bone metastases, where cancer cells exploit the normal bone remodeling process and shift the balance towards increased bone resorption. Parathyroid hormone-related protein (PTHrP), MMPs and PGE2 are some of the factors released by tumor cells that modulate the expression of receptor activator of NF-κB ligand (RANKL) by osteoblasts, which is a master regulator of osteoclast differentiation^65,66^. Increased RANKL production by osteoblasts and osteocytes in turn enhances osteoclast differentiation and activity, leading to extensive bone degradation. On the other hand, bone matrix-embedded factors released during resorption, such as TGF-β, insulin growth factor, and platelet-derived growth factor, further stimulate tumor growth and perpetuate a “vicious cycle” of bone destruction^67^. Biphosphonates and denosumab (an anti-RANKL human monoclonal antibody) are commonly used as adjuvant therapies for the treatment of metastatic bone disease to normalize the level of osteoclastic activity^68^. However, although these treatments alleviate skeleton-related symptoms, new and more effective therapeutic targets are needed to suppress the establishment of the vicious cycle.

### The SNS and bone metastatic disease

Bones are highly innervated organs, with a high density of sensory and sympathetic nerve fibers in the periosteum and along blood vessels in the bone marrow^69^. A physical and functional association of nerve fibers and bone cells is to be expected^70^, since the nerve fiber density is usually increased near surfaces with enhanced bone turnover^71^.

Although cells of osteoblast and osteoclast lineages have been reported to express α-AR mRNA, its relative expression compared to that of the β_2_-AR subtype is greatly reduced^72–74^. β_2_-AR but not β_1_-AR or β_3_-AR is widely expressed in primary osteoclasts and osteoclastic cell lines^5,75^, as well as in osteoblast lineage cells^76–78^. β_2_-AR is fully functional in bone cells, since β_2_-AR agonism triggers an increase in intracellular cAMP in vitro^77^. Interestingly, cells of the osteoblast lineage also express monoamine oxidase (MAO)α and MAOβ^79^, as well as the NE transporter^80^, and are thus able to take up and catabolize NE from the external milieu.

**Fig. 3:**
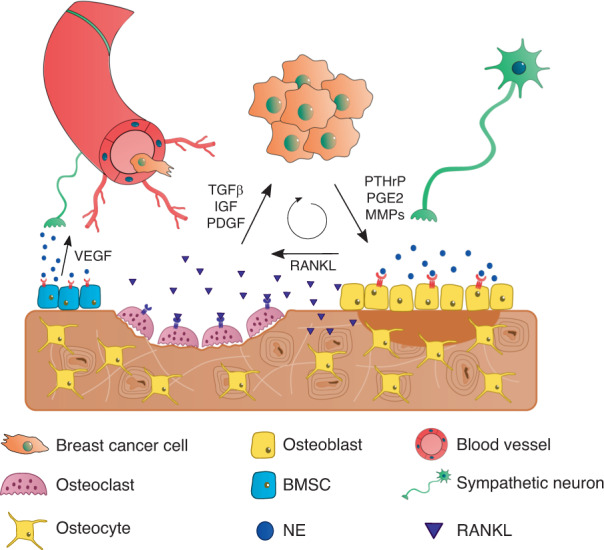
The bone metastatic niche and the metastatic vicious cycle. Once engrafted in the bone, breast cancer cells secrete pro-osteoclastic factors such as PTHrP, PGE2, and MMPs, which induce the expression of RANKL by osteoblasts and osteocytes, promoting osteoclast differentiation and activity. In turn, factors released from the bone matrix enhance the growth of cancer cells, establishing a metastatic vicious cycle that leads to extensive bone degradation. NE, norepinephrine; BMSC, bone marrow stromal cell

β-AR activation in bone triggers osteoclastic differentiation, diminished bone formation and consequent bone loss (reviewed in^81^), mostly due to an increase in RANKL production by osteoblast lineage cells in vivo^82,83^ (Fig. 3). Similarly, β_2_-AR agonism was reported to increase RANKL production by the MLO-Y4 osteocytic cell line in vitro and consequently to induce the differentiation of the RAW264.7 osteoclastic cell line in coculture experiments^84^. Although osteocytes have received increasing attention in recent years regarding their role in the modulation of breast cancer progression^85–88^, data on the action of adrenergic signaling pathways on osteocytes in this context are still scarce. Osteocytes express β_2_-AR, and as they are the most common cell type in bone, the importance of their putative crosstalk with the SNS in breast cancer should not be overlooked. Regardless, SNS activation of osteoblast-lineage cells seems to further potentiate the establishment of a metastatic vicious cycle upon bone metastatic colonization of breast cancer.

Campbell and colleagues have made important contributions to this field of research. In a mouse model of bone metastasis established by intracardiac injection of bone-tropic MDA-MB-231 cells, the authors showed that adrenergic stimulation of the bone stroma potentiated the establishment of the metastatic vicious cycle^11^. Chronic immobilization stress, as a model of endogenous sympathetic activity, was used to demonstrate that augmented catecholamine levels led to the formation of larger osteolytic lesions, an effect mediated by β_2_-AR^11^. Moreover, isoproterenol administration before injection of breast cancer cells increased the numbers of tumor foci and lesions in bone, suggesting that sympathetic triggering in the bone microenvironment facilitated breast cancer cell engraftment. The authors suggested that this effect was partially due to RANKL signaling and its chemotactic action on MDA-MB-231 cells^11^.

**Fig. 4:**
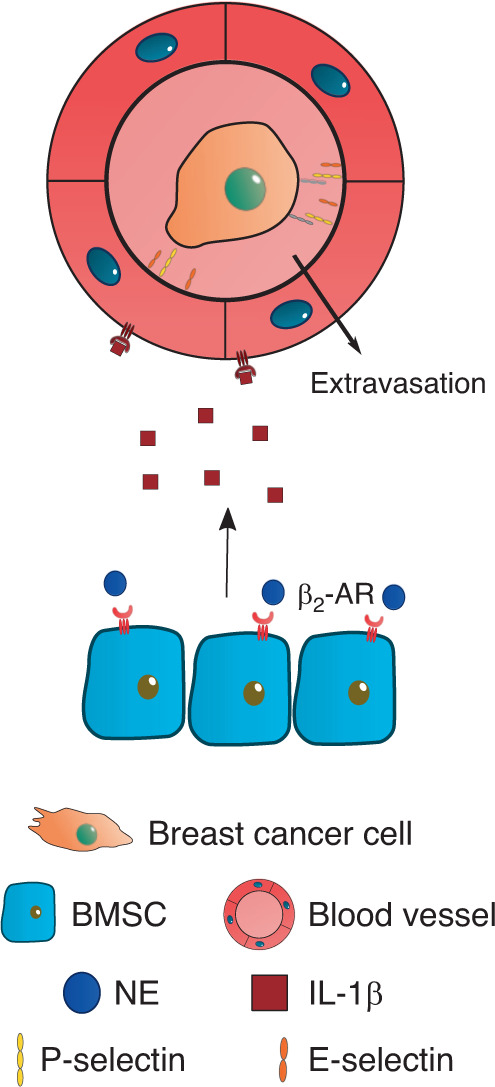
Breast cancer cell extravasation into the bone niche. NE stimulation of stromal β_2_-AR is associated with an increased release of VEGF and IL-1β, which leads to augmented angiogenesis and expression of P- and E-selectins in endothelial cells. The latter event promotes breast cancer cell extravasation from the circulation into the bone marrow

In addition to augmented RANKL signaling, adrenergic stimuli promoted breast cancer extravasation and retention in the bone through modulation of the bone vasculature. Nude mice subjected to either chronic immobilization stress or isoproterenol administration showed increased VEGF-A expression by bone marrow stromal cells (BMSCs) and consequent angiogenesis, which resulted in the promotion of breast cancer cell colonization^89^. Furthermore, incubation of BMSCs with isoproterenol led to the release of IL-1β, which in turn activated E/P-selectin expression in endothelial cells and enabled the adhesion and retention of breast cancer cells in vitro^90^ (Fig. 4).

Interestingly, the interplay between the SNS and breast cancer in the bone metastatic niche is not unidirectional. Not only is the SNS capable of inducing breast cancer cell engraftment and proliferation through RANKL and VEGF-A signaling, but conversely, breast cancer may also be able to regulate AR dynamics in the bone niche. Breast cancer cell-secreted PTHrP is a well-known modulator of bone turnover in the metastatic niche (reviewed in^91^). PTHrP binds to PTH receptor 1 (PTHR1) expressed in osteoblasts and upregulates RANKL expression to promote osteoclastogenesis, driving the vicious metastatic cycle^92^. Interestingly, PTHR1, β_2_-AR, and their corresponding downstream pathways in osteoblastic cells seem to be intimately connected. Using germline β_2_-AR knockout mice, Hanyu et al. demonstrated that β_2_-AR expression is required for the osteoanabolic effect of PTH and that β_2_-AR modulates the expression of PTHR1 target genes, such as RANKL, alkaline phosphatase, bone sialoprotein, and osteoprotegerin (a RANKL decoy receptor), in osteoblasts^93^. On the other hand, PTH was shown to directly downregulate β_2_-AR expression in osteoblast-like MC3T3-E1 cells in vitro^94^. This interdependency might be explained by common intracellular downstream effectors that are triggered by binding of their corresponding ligands. Both PTHR1 and β_2_-AR are GPCRs that signal through the adenylyl cyclase/PKA axis and promote the phosphorylation of cAMP-response element binding protein to induce transcription of target genes^94^. Furthermore, after ligand binding, both receptors are rapidly desensitized through pathways dependent on β-arrestin and β-adrenergic kinase 1^95–97^, which can also act as protein scaffolds that subsequently lead to the activation of the mitogen-activated protein kinases ERK1/2 and several other effector molecules^98^. However, while these interactions have been described to occur between PTHR1 and β_2_-AR in the context of intermittent PTH treatment, it is still unknown whether breast cancer-secreted PTHrP can elicit the same response in the context of bone metastatic disease. Although PTHrP and PTH share the same receptor, there are several described noncanonical pathways for the action of PTHrP whose importance is still poorly understood^91^. Therefore, more data on the interplay between PTHrP and β_2_-AR in breast cancer bone metastasis are urgently required, since this knowledge could change our understanding of the dynamics of β_2_-AR expression in bone throughout the progression of this disease and facilitate the design of new, more effective therapeutic options.

## Breast cancer and beta-blockers: a clinical perspective

Although preclinical data are extremely valuable for understanding the many processes that control breast cancer progression and metastatic spread, it is crucial to translate the results into a clinical setting. In the past decade, increasing attention has been devoted to the effect of sympathetic activity on breast cancer patient survival and breast cancer recurrence^10,99^. In this section, we will review the published epidemiologic and clinical data on the effect of several β-AR antagonists (henceforth called beta-blockers) on breast cancer and discuss the limitations associated with the interpretation of the reported results.

Epidemiologic studies have previously suggested that patients with cancer subjected to high levels of psychosocial stress usually have a poorer prognosis and survival than those not subjected to these conditions^100^. SNS-targeting beta-blockers are thus potential therapeutic options for cancer and are already widely used in other pathological settings, such as the treatment of asthma and hypertension^101,102^. The safety profile of these drugs is well described, and they are not associated with an increased incidence of breast cancer, as evidenced by previous epidemiologic studies^103,104^.

A proof-of-principle study performed by Powe et al. analyzed the effect of beta-blocker prescription prior to breast cancer diagnosis on patient survival^10^. Reduced tumor recurrence and metastasis incidence and increased patient survival rates were reported in the beta-blocker-treated group, with no significant differences in tumor stage, tumor size, tumor grade or vascular invasion between the treated and placebo groups.

However, the population size in that study was relatively small, and no distinction between the type of beta-blockers used was included in the analysis^10^. Atenolol and bisoprolol are β_1_-AR specific, while propranolol and timolol are nonspecific β_1/2_-AR antagonists; therefore, the contributions of the different ARs to the reported results cannot be isolated. In fact, another population-based study by Barron et al. showed a beneficial effect of propranolol but not atenolol on breast cancer metastasis and patient survival^105^. Interestingly, Melhem-Bertrand et al. reported a beneficial effect of the β_1_-AR-targeting drugs metoprolol and atenolol on the recurrence of triple-negative breast cancer (TNBC) but not on ER-positive breast cancer, highlighting the importance of breast cancer receptor status on the response to beta-blockers^106^. Thus, it is still unclear which receptors are the main contributors to the reported beneficial effects of beta-blockers on breast cancer recurrence, and this topic is a matter of intense debate. However, we hypothesize that a broader acting beta-blocker, such as propranolol, could be even more beneficial than specific beta-blockers in managing breast cancer recurrence and metastasis.

Several studies have suggested that beta-blocker usage could be explored as an adjuvant therapy in breast cancer treatment^10,29,105–109^. However, these studies have some limitations, such as a retrospective design, small population size, difficulties in the assessment of beta-blocker treatment duration and compliance, or a lack of access to data on comorbidities and other medications. Other retrospective studies reported no correlation between beta-blocker usage and reduced breast cancer-specific mortality or recurrence^99,110–112^, and thus, the benefits of these drugs remain controversial (for more details, refer to Table 2). Randomized clinical trials are warranted to assess the clinical relevance of beta-blockers for breast cancer treatment.

**Table 2:** Summary of epidemiologic studies regarding the influence of β-blockers on breast cancer outcomes

Treated group size/total size	β-Blocker used (population size)	Improved patient survival (HR; CI)	Reduced tumor recurrence (HR; CI)	Reduced incidence of metastasis (HR; CI)	Reference
43/466	Atenolol (25)	Yes (0.291; 0.119–0.715)	Yes (−)	Yes (0.430; 0.200–0.926)	^10^
	Propranolol (7)				
	Bisoprolol (7)				
	Timolol (4)				
595/4 738	Atenolol (525)	Yes (0.19; 0.06–0.60)	N.D.	Yes (−)	^105^
	Propranolol (70)				
204/1 779	Atenolol (−)	No (0.76; 0.44–1.33)	No (0.86; 0.57–1.32)	N.D.	^134^
	Metoprolol (−)				
	Propranolol (−)				
	Others (−)				
102/1 413	Metoprolol (43)	No (0.64; 0.38–1.07)	Yes (0.52; 0.31–0.88)	N.D.	^106^
	Atenolol (38)				
	Others (21)				
74/800	Carvedilol (11)	Yes (0.42; 0.18–0.97)	Yes (0.52; 0.28–0.97)	Yes (0.32; 0.12–0.90)	^108^
	Sotalol (3)				
	Atenolol (27)				
	Betaxolol (1)				
	Bisoprolol (11)				
	Metoprolol (8)				
	Nebivolol (13)				
3 660/18 733	Metoprolol (1 793) Atenolol (622)	N.D.	No (1.3; 1.1–1.5)	N.D.	^111^
	Propranolol (586)				
	Others (659)				
1 770/55 252	Propranolol (1 770)	No (0.94; 0.77–1.16)	N.D.	N.D.	^99^
1 443/5 754	Carvedilol (22)	No (1.11; 0.94–1.32)	N.D.	N.D.	^112^
	Sotalol (84)				
	Atenolol (854)				
	Bisoprolol (189)				
	Metoprolol (45)				
	Propranolol (249)				
153/1 144	Bisoprolol (59)	No (1.05; 0.85–1.29)	Yes (0.81; 0.66–0.99)	N.D.	^109^
	Metoprolol (48)				
	Atenolol (28)				
	Propranolol (13)				
	Others (5)				
93/956	N.D.	Yes (0.48; 0.23–0.99)	No (0.93; 0.39–2.25)	Yes (0.40; 0.17–0.93)	^29^

*HR* hazard ratio, *CI* 95% confidence interval (lower limit–higher limit), *N.D.* no data

To our knowledge, the only results from phase II placebo-controlled clinical trials published to date address the effect of perioperative propranolol administration on several metastatic biomarkers in patients with early breast cancer. Zhou et al. reported decreased immunosuppression after the administration of propranolol compared to placebo controls during the perioperative period of breast cancer surgery^113^. Propranolol was also shown to block the proliferation of patient-derived regulatory T cells^113^. Shaashua^114^ and Haldar^115^ reported a reduction in the expression of EMT-related genes in resected primary tumors from patients simultaneously treated with propranolol and the COX-2 inhibitor etodolac. The resected tumors also showed reduced expression of prometastatic, antiapoptotic and proliferation markers; increased infiltration of B-cells; and a decreased population of TAMs. Propranolol- and etodolac-treated patients also presented reduced levels of the circulating inflammatory cytokines IFNγ and IL-6 and increased levels of NK cell activation during treatment^114^. Another randomized clinical trial by Hiller et al. showed similar results with the administration of propranolol for one week before surgical resection of the primary breast tumor^116^. In this study, compared to placebo-treated controls, patients treated with propranolol before surgery showed reduced EMT gene expression and increased dendritic cell infiltration and M1 macrophage polarization in the resected tumors. Interestingly, compared to clinically responsive patients, patients clinically nonresponsive to propranolol (i.e., without significant reductions in blood pressure and heart rate after beta blockade) showed decreased tumor EMT gene expression, although immune cell infiltration in the primary tumor was changed^116^. These clinical trials pointed to a possible beneficial effect of propranolol on reducing the metastatic potential of primary breast tumors. However, adequately powered clinical trials with a focus on overall survival and cancer recurrence are still needed before propranolol can be used for breast cancer treatment.

## Conclusion and future perspectives

Despite the advances made in recent years, knowledge on the impact of endogenous stress on the complex interactions governing breast cancer disease progression is still incomplete. This review summarizes and combines the available data regarding SNS signaling in the orchestration of breast cancer.

To date, adrenergic signaling has been implicated in several steps of disease progression, promoting tumor growth, angiogenesis, immunosuppression and invasion (Fig. 1). While several in vitro studies and animal models have illustrated the intricate control exerted by the SNS over cancer cellular processes, the contributions of the different ARs expressed in the multiple cellular components of the tumor microenvironment remain puzzling. Furthermore, the inherent heterogeneity of breast cancer presents an additional challenge in modeling this disease. The distinctive AR expression patterns in breast cancer cell lines widely used in the various experimental models are certainly relevant, and more information on the adrenergic control of disease progression in different cell lines is urgently needed.

Modeling the various cellular and structural components of the cancer niche is still technically challenging. The use of immunodeficient mice is required for xenograft models, but the contribution of the immune system is not considered in these models. Thus, current in vitro and in vivo models do not completely recapitulate the complexity of the disease, but as new, more complicated models are developed, discerning the specific contributions of each cell type becomes increasingly difficult. Specific deletion of β_2_-AR in not only breast cancer cells^46^ but also osteoblasts^89^ and macrophages^117^ could be used as an important tool to elucidate the role of this receptor in various models of the disease, although no models of conditional β_2_-AR knockout specifically in osteoclasts or osteocytes have been described to date. Furthermore, microfluidic systems have several advantages when compared to traditional in vitro models since they allow the compartmentalization of different cell types and the introduction of fluid flow, which can be physiologically relevant. Microfluidic platforms have already been developed for the study of breast cancer metastasis to bone^118–121^, but modeling the SNS in these platforms is still challenging.

Metastatic tropism for bone is an evident feature of breast cancer, and bone is the most common site of metastasis in luminal breast cancer patients^52^. Although adrenergic stimulation of the bone microenvironment is thought to increase osteolysis and potentiate the metastatic vicious cycle, the SNS-controlled interactions between breast cancer and bone cells remain mostly unexplored, apart from the contributions of Elefteriou and his group^11,89,90^. Although the use of luminal A breast cancer cell lines in bone metastasis models presents technical challenges due to the less invasive phenotype of these cell lines, it is crucial to understand the molecular changes that might be elicited by the SNS in these cells. Furthermore, since luminal A tumors are the most common subtype of breast tumors in patients, the use of luminal subtype breast cancer cells in in vitro and in vivo models of this disease is certainly more clinically relevant than the currently widespread use of aggressive TNBC cells.

Future developments in novel targeted therapeutic strategies, such as tumor-specific denervation via viral vectors^34^, are exciting fields of research that will require input from various areas of expertise before becoming applicable in a clinical setting. It is still unclear whether this technique can be applied to locally and specifically denervate bone in preclinical studies. In addition, other denervation techniques, such as chemical sympathectomy by local delivery of guanethidine into the femoral bone marrow via an osmotic minipump, have been established^122^, which could help to clarify the role of sympathetic nerves in bone metastasis.

Finally, clinical observations on the usage of beta-blockers for the treatment of breast cancer suggest that interfering with SNS signaling could have beneficial effects on patients, particularly in the control of metastatic spread. However, systemic administration of beta-blockers can also have unforeseen consequences on the progression of breast cancer, and adequately powered clinical trials are needed before their therapeutic implementation. Targeted drug delivery systems could address the currently unmet clinical challenge of circumventing the disadvantages of systemic beta-blocker administration. The unique biochemical and biophysical characteristics of the bone microenvironment provide the means for targeted drug delivery to bone metastatic tumors. Bisphosphonates^123^, acidic amino acid peptidic sequences^124^, liposomes^125^, organic^126^, and inorganic^127^ nanoparticles, chimeric peptides targeting CatK^128^ and HER2-targeting nanoparticles^129^ have been previously used to achieve bone metastasis-specific drug and gene delivery in vivo. Whether these strategies can be used to deliver SNS-targeting drugs specifically to the bone microenvironment and whether they can be translated into a clinical benefit remain to be elucidated.

Taken together, the data summarized in this review highlight the importance of SNS activation in breast cancer. In the next few years, exciting new developments are expected that would allow us to complement our understanding of the molecular cues that drive breast cancer progression.
